# Availability of adequate iodized salt at household level in rural communities in Farta district, Northwest, Ethiopia: a cross-sectional study

**DOI:** 10.1186/s40795-023-00728-7

**Published:** 2023-06-23

**Authors:** Yalelet Fentaw Shiferaw, Wubshet Debebe Negash, Daniel Gashaneh Belay, Haileyesus Birhan, Desale Bihonegn Asmamaw

**Affiliations:** 1 Department of Nutritional Care and Counseling, University of Gondar Specialized Hospital, Gondar, Ethiopia; 2 Department of Health Systems and Policy, Institute of Public Health, College of Medicine and Health Sciences, University of Gondar, Gondar, Ethiopia; 3 Department of Human Anatomy, College of Medicine and Health Sciences, University of Gondar, Gondar, Ethiopia; 4 Department of Epidemiology and Biostatistics, Institute of Public Health, College of Medicine and Health Sciences, University of Gondar, Gondar, Ethiopia; 5 Department of Reproductive Health, Institute of Public Health, College of Medicine and Health Sciences, University of Gondar, Gondar, Ethiopia

**Keywords:** Iodized salt, Household level, Farta district, Ethiopia

## Abstract

**Introduction:**

Iodine is a crucial trace element for thyroid hormone synthesis. All age groups are affected by iodine deficiency disorders (IDD), especially pregnant women, young women, and children. Iodine deficiency disorder has been recognized as a serious public health issue in Ethiopia. Therefore, the aim of the current study was to assess the availability of iodized salt and associated factors at the household (HH) level in the Farta district, northwest, Ethiopia.

**Methods:**

A community-based cross-sectional study was conducted involving 704 participants in Farta District, Northwest Ethiopia, between February and March 2021. A multi-stage sampling technique was employed to recruit participants. Data were collected using pretested and structured questionnaires administered by face-to-face interviewers. The data were entered into Epi-data version 4.6 software and exported into Stata version 14 for the purpose of analysis. Both bivariable and multivariable logistic regression analyses were used to identify factors associated with the availability of iodized salt. Statistical significance was declared at a p-value ≤ 0.05 with a corresponding 95% confidence interval.

**Results:**

The availability of adequately iodized salt in the study area was 26.6% (CI = 23.8%, 29.4%) of households. Good knowledge about iodine deficiency disorder (IDD) and iodized salt (AOR = 3.85, 95% CI: 3.65, 6.11); short-term storage of salt at the HH [AOR = 2.76, 95% CI: 1.98, 3.23); and stored in covered containers (AOR = 1.57, 95% CI: 1.11, 1.78) were significantly associated with the outcome variable.

**Conclusion:**

The availability of adequate iodized salt at the HH level was far below the WHO target (more than 90%) in the study area. Knowledge about IDD and iodized salt, stored in covered containers, and short-term storage of salt at the HH were significantly associated with the availability of iodized salt. Hence, at the household level, increasing awareness and disseminating information about the benefits of using iodized salt could be effective. Moreover, education about the proper storage and handling of iodized salt, as well as the recommended storage duration of salt in the home, should be provided as part of our community outreach programs.

**Supplementary Information:**

The online version contains supplementary material available at 10.1186/s40795-023-00728-7.

## Introduction

In order to synthesize thyroid hormone (TH), iodine is required. It regulates the body’s growth, development, reproduction, and metabolism [1]. Humans suffer from iodine deficiency disorders (IDDs) when they do not receive enough iodine on a daily basis [2]. These disorders can lead to endemic goiters, hypothyroidism, reproductive problems, or dwarfism. Iodization of salt is considered one of the most cost-effective, widely used, and low-cost interventions for reaching the general population with iodine [1, 2].

Iodine deficiency affects less than 10% of the world’s population [3]. IDDs affect people of all age groups, though young children, reproductive-age women, pregnant women, and postpartum women are most vulnerable [4]. There is still a public health concern regarding IDDs. Several studies have shown that households in South Asia and sub-Saharan Africa don’t have access to iodized salt [5]. According to Ethiopian pocket-area studies, many households used insufficient amounts of iodized salt compared to the universal standard [6–8]. Nevertheless, according to the 2016 Ethiopian Demographic Health Survey, 89% of HHs consume enough iodized salt [9].

A number of factors determine the availability of iodized salt, such as using packed salt, not exposing salt to sunlight, storing salt in a dry area, storing the salt for a short period of time, adding salt late after cooking, and knowing about iodized salt and IDDs [6–8, 10]. In the past, iodine content was evaluated qualitatively using a rapid test kit, but it was not reliable (low specificity). Titration with iodometry (IDTM) is the most efficient and preferred method. In order to determine salt’s iodine content, this method is reliable [11–13].

In order to prevent IDD, salt should contain sufficient iodine content at the HH level. It is important to give attention to how much-iodized salt should be stored [14, 15]. According to the literatures, iodine is highly volatile and will be lost during cooking due to high humidity and boiling. Even if the effect is small, light or dry heat causes iodine to be lost from the salt [14–16]. In Ethiopia, there is a lack of consistency in findings, and no studies have been conducted using the titration method in the study area. Furthermore, the current study was conducted in highland areas, that is, areas with a high prevalence of goiter, whereas the previous studies were conducted in lowland areas [17–21]. Hence, this study used the gold standard IDTM to assess the prevalence of adequately iodized salt usage and associated factors in Farta district, Northwest Ethiopia.

## Methods

A community-based cross-sectional study was conducted in Farta district, northwest Ethiopia, from February to March 2021. The district has 34 kebeles (the lowest administrative unit), and it is 671 km from Ethiopia’s capital, Addis Ababa. Nearly half of the district’s population (49.7%) is female. A total of 23 health posts and six health centers serve the catchment area’s residents.

### Study participants

The source population was comprised of all households in the Farta district. The study population was comprised of all households in the selected kebeles of the Farta district. Individuals with severe illnesses and those unable to prepare food at home were excluded from the study.

### Sample size determination and sampling procedures

Based on a single population proportion formula, a sample size of 704 was calculated, taking into account the following statistical assumptions: a margin of error of 5% (0.05), a Z-value of 1.96, 10% non-response rates, a design effect of 2, and 29.7% of the proportion of availability of iodized salt at HH level (22).

We used a multistage sampling technique stratified by kebeles in urban and rural areas. Using simple random sampling, 10 rural kebeles and 1 urban kebele were selected from the 32 rural and two urban kebeles found in the district. Based on the household identification numbers in the health extension workers’ registration book (family folders), a sampling frame was prepared. Frames containing study subjects were formed for each selected kebele using the household identification numbers. After that, the proportional allocation technique was used to determine the study participants from each kebele. From the existing sampling frame, households were selected using a simple random sampling technique (Open Epi Random Program version 3).

### Laboratory procedures

IDTM was done to determine the iodine content in the salt. From each systematically selected household, a 50 g mixed (homogenized) salt sample was taken using a moisture-free, clean, airtight plastic container. The sample was labeled and coded with the date of sampling, source of salt, and batch number. To calculate the iodine concentration, each sample was analyzed in triplicate, and the average sample concentration was used. Sulphuric acid, potassium iodate, and potassium iodide are used as principal reagents, standardized sodium thiosulphates are used as a titrant, and the starch solution is used as an indicator. Based on the titration results, the samples were classified according to their iodine content. The outcome variable, adequately iodized salt utilization measured as parts per million (ppm) < 15 was considered inadequately iodized, while ≥ 15 ppm is considered adequately iodized. The test was carried out in an Ethiopian public health institution’s laboratory [22]. Knowledge about IDD and iodized salt was categorized as “poor,“ “average,“ and “good” for those respondents who scored less than 3, 3–5, and greater than or equal to 6 from nine questions, respectively. The duration of storage was operationalized as less than or equal to two months being considered short-term storage of salt at the household level, while those who stored greater than two months were considered to be in long-term storage [23].

### Data collection tools and quality control

Based on a review of related literature conducted in different regions of Ethiopia, a structured interviewer-administered questionnaire was developed [1, 24–26]. Data collection was carried out by eight female BSc midwives (data collectors) and two supervisors with experience in research and fieldwork coordination. Orientation and training were provided for two days to data collectors and supervisors on how to interview and use bias-control mechanisms. Pretests were conducted on 35 study participants (5%) in the Fogera district, and modifications were made based on the results.

### Data processing and analysis

We manually checked the collected data for completeness and consistency. For cleaning, coding, and analysis, the data was imported into Epi-data version 4.6 and exported to Stata version 14. Frequencies, percentages, means, and standard deviations were used to describe descriptive statistics. The distribution of continuous variables was examined using normality tests, such as kurtosis and skewness.

Using variance inflation factors (VIF), we checked for multicollinearity among independent variables; one of them (exposure to sunlight) had VIF values of greater than 10, so it was excluded from the analysis [27]. We conducted a binary logistic regression analysis. In the multivariable model, independent variables with a p-value of 0.2 were considered. A p-value less than 0.05 was considered statistically significant. To examine the strength and direction of the association, the AOR was calculated with its 95% CI.

## Results

In total, 704 households responded to the study, with a 100% response rate. The average age of respondents was 31.5 years, with a standard deviation ± 5.02. More than one-third of the respondents (36.3%) were between the ages of 18 and 29. Most of the study participants did not have formal education (70.9%) and lived in rural areas (85.1%). There were 434 respondents (61.6%) who were married. Regarding salt containers, 571 people (81.1%) stored their salt in covered containers. Over two-fifths of the study participants (41.3%) had good knowledge of IDD and iodized salt (Table 1).

**Table 1:** Sociodemographic and other related factors in the HH level in Farta distrct, northwest, Ethiopia

Variables	Frequency (N)	Percentage (%)
Place of residence		
Rural	599	85.1
Urban	105	14.9
Education of the participant		
No formal education	499	70.9
Formal education	205	29.1
Marital status		
Currently married	434	61.6
Unmarried	270	38.4
Age in years		
18–29	256	36.3
30–44	237	33.7
45–65	211	30
Salt container		
With cover	571	81.1
Not cover	133	18.9
Knowledge		
Good	291	41.3
Average	229	32.5
Poor	187	26.2
Duration of storage in months		
≤ 2 months	320	45.5
> 2 months	384	54.5
Place of storage		
Dry area	589	83.7
Moist and hot area	115	16.3
Distance travel to buy salt		
≤I hour waking	515	73.1
>I hour waking	189	26.9
Exposed to sunlight		
Yes	169	24
No	535	76

### Associated factors of adequate iodized salt

According to the current study, 26.6% of respondents had adequately iodized salt (CI = 23.8%, 29.4%). The median iodine content of the sampled salt was 16.26 ppm, with an interquartile range of 9.87 ppm to 25.39 ppm. Households with good knowledge of IDD were 3.85 times more likely to have iodized salt than those with poor knowledge (AOR = 3.85, 95% CI: 3.65, 6.11). Compared to the participants who did not store their salt in a covered container, those who did have a 1.57-fold higher likelihood of having adequately iodized salt (AOR = 1.57, 95% CI: 1.11, 1.78). The odds of having adequately iodized salt were 2.76 times higher among those who stored ≤ 2 months after purchasing as compared to their counterparts (AOR = 2.76, 95% CI: 1.98, 3.23) (Table 2).

**Table 2:** Factors associated with adequate iodized salt at HH levels in Farta district, Northwest, Ethiopia

Variables	Availability of iodized salt in household level		COR (95% of CI)	AOR (95% of CI)
	≥ 15 ppm	< 15ppm		
Education of the participant				
No formal education	133	366	1	1
Formal education	52	153	0.94 (0.59, 1.71)	1.25 (0.98, 1.43)
Marital status				
Currently married	122	312	1.28 (0.85, 2.19)	1.21 (0.89, 1.48)
Unmarried	63	207	1	1
Age in years				
18–29	79	177	1.74 (0.95, 2.35)	1.55 (0.97, 2.14)
30–44	63	174	1.42 (1.02, 1.98)	1.33 (0.89, 1.76)
45–65	43	168	1	1
Salt container				
With cover	157	414	1.42 (1.03, 1.89)	1.57 (1.11, 1.78)
Not cover	28	105	1	1
Knowledge				
Good	106	185	4.09 (3.79, 6.21)	3.85 (3.65, 6.11)
Average	56	173 3979	2.31 (1.16, 3.23)	1.85 (0.98, 2.37)
Poor	23	164	1	1
Duration of storage in months				
≤ 2 months	119	201	2.85 (1.91, 3.87)	2.76 (1.98, 3.23)
> 2 months	66	318	1	1
Place of storage				
Dry area	166	423	1.98 (1.12, 2.68)	1.54 (0.93, 2.79)
Moist and hot area	19	96	1	1
Distance travel to buy salt				
≤I hour waking	146	369	1.52 (0.71, 3.57)	1.41 (0.60, 2.24)
>I hour waking	39	150	1	1

## Discussion

This study showed that 26.6% of households had adequately iodized salt; this study was consistent with a study done in Gondar town, northwest Ethiopia [28], but this was lower than those found in studies from Ethiopia [1, 13, 19], Saudi Arabia [29], Bangladesh [29], Nepal [24]. This difference may be explained by using IDTM in the current study, different study populations and study settings, poor community awareness of the prevention of IDDs, and the benefits of iodized salt [8, 24, 28].

In this study, the odds of having adequately iodized salt at a HH level were higher among participants who had good knowledge about IDD and iodized salt utilization compared to those who had poor knowledge. This finding is consistent with the studies done in Ethiopia [7] and Ghana [13]. The reason for this may be that those who had good knowledge of IDD and iodized salt stored it properly, handled the salt properly, and followed the recommended salt storage duration [13].

Furthermore, participants who stored salt in covered containers had a higher chance of having adequately iodized salt at the HH level than those who did not. This study is supported by studies in Ethiopia [30, 31]. This could be due to the fact that the iodine content of the salt may be retained or maintained in the covered container [26]. Moreover, salt in a covered container is not exposed to sunlight [31].

The odds of adequate iodized salt usage were more likely among those who stored ≤ 2 months after purchasing as compared to their counterparts. Which is in line with the studies done in northern Ethiopia [13, 28]. While being stored and distributed, iodine becomes easily volatile when exposed to heat, humidity, moisture, and light [32]. Furthermore, iodized salt loses 28–51% of its iodine after three months, 35–52% after six months, and up to 66% after 12 months. Iodized salt lost 30-98% of its original iodine content due to high humidity levels [28, 32].

This finding show only one-fourth of households had adequate amounts of iodized salt in their homes, which implies the majority of the population suffers from IDDs. Because of IDDs, endemic goiter, hypothyroidism, reproductive failure, or dwarfism can occur. Therefore, it is crucial to counteract the problem by working to increase the availability of iodized salt at the household level. This study has its own limitation, the study was done in one region of the highland, and the findings may not be generalizable to national levels. The cross-sectional nature of the study makes it impossible to determine cause-and-effect relationships. Further, there was no triangulation with qualitative research in this study.

## Conclusion

In conclusion, the availability of adequate iodized salt at the HH level was far below the WHO target (more than 90%) in the study area. Knowledge about IDD and iodized salt, stored in covered containers, and short-term storage of salt at the HH were significantly associated with the availability of iodized salt. Hence, at the household level, increasing awareness and disseminating information about the benefits of using iodized salt could be effective. Moreover, education about the proper storage and handling of iodized salt, as well as the recommended storage duration of salt in the home, should be provided as part of our community outreach programs.

## Electronic supplementary material

Below is the link to the electronic supplementary material.

Supplementary Material 1

## Data Availability

All the data generated in this study are included in this manuscript. The datasets used and/or analyzed to produce the current manuscript will be obtained from the corresponding author whenever required.
