# Supplementary material for: Availability of adequate iodized salt at household level in rural communities in Farta district, Northwest, Ethiopia: a cross-sectional study
Source: BMC Nutr. 2023 Jun 23;9:71. doi: 10.1186/s40795-023-00728-7 (PMC10288757; doi:10.1186/s40795-023-00728-7)
Supplement: Supplementary file 1 — Supplementary Material 1 [file 40795_2023_728_MOESM1_ESM.docx]

**English version questionnaires**

Questionnaire code ________

Kebele_________

Section I: Socio-demographic characteristics of the participants

| NO. | Questions | Response | Skip |
| --- | --- | --- | --- |
| 101 | Age of the participants? | --------in year |  |
| 102 | What is your usual place of residence area | 1=rural  2=urban |  |
| 1043 | Educational status of the participant? | 1=unable to read and write  2=able to read and write  3=primary school  4=secondary school  5=college and above  6=other(specify----) |  |
| 104 | What is your current marital status? | 1=Single  2=Married  3=Widowed  4= Divorced  5=Separated  6=other (specify….) |  |
| **Section II: knowledge and iodine related questions** | | | |
| 201 | Have you ever heard of iodized salt | 1=Yes  2=No |  |
| 202 | Do you know the importance of iodized salt for human health? | 1=yes  2=no |  |
| 203 | Do you know the consequence of iodine deficiency? | 1=Yes  2=No | If no, skip question number 205 |
| 204 | What are the effects of iodine deficiency you know | 1= goiter  2 = effect on child learning capacity  3= stillbirth  4= effect on growth and development of the body  5= other (specify…) |  |
| 205 | Do you thing that every salt contains iodine | 1= yes  2= no |  |
| 206 | At what time does iodized salt is to be added during food cooking | 1=early and at the middle of the cooking  2= right after cooking |  |
| 207 | What type of care needed for salt? | 1= far from heat and faire  2= prevent from moisture  3=prevent from sunlight  4=Others |  |
| 208 | Do you know iodized salt have the level of iodine and expiry date in their container? | 1=yes  2=no |  |
| 209 | Do you think if the storage of iodized salt kept near heat loss its iodine content? | 1= yes  2=no |  |
| 210 | Duration of storage in month | _______ |  |
| 211 | Place of storage | 1=dry area  2=moist and hot areas |  |
| 212 | Distance travel to buy salt | ____in hour |  |
| 213 | Exposed to sunlight | 1=Yes  2=No |  |
| 214 | How do you sore the salt | 1= container with cover  2= container without cover |  |
